# Creep-Based Reliability Evaluation of Turbine Blade-Tip Clearance with Novel Neural Network Regression

**DOI:** 10.3390/ma12213552

**Published:** 2019-10-29

**Authors:** Chun-Yi Zhang, Jing-Shan Wei, Ze Wang, Zhe-Shan Yuan, Cheng-Wei Fei, Cheng Lu

**Affiliations:** 1 School of Mechanical and Power Engineering, Harbin University of Science and Technology, Key Laboratory of Advanced Manufacturing and Intelligent Technology, Ministry of Education, Harbin 150080, China; zhangchunyi@hrbust.edu.cn (C.-Y.Z.); weijingshan_ma17@hrbust.edu.cn (J.-S.W.); wangze_ma17@hrbust.edu.cn (Z.W.); yuanzheshan_ma17@hrbust.edu.cn (Z.-S.Y.); 2 Department of Aeronautics and Astronautics, Fudan University, Shanghai 200433, China; 3 School of Aeronautics, Northwestern Polytechnical University, Xi’an 710072, China; lucheng2013@163.com

**Keywords:** blade-tip radial running clearance, high-temperature creep, generalized regression extremum neural network, distributed collaborative response surface method, reliability analysis

## Abstract

To reveal the effect of high-temperature creep on the blade-tip radial running clearance of aeroengine high-pressure turbines, a distributed collaborative generalized regression extremum neural network is proposed by absorbing the heuristic thoughts of distributed collaborative response surface method and the generalized extremum neural network, in order to improve the reliability analysis of blade-tip clearance with creep behavior in terms of modeling precision and simulation efficiency. In this method, the generalized extremum neural network was used to handle the transients by simplifying the response process as one extremum and to address the strong nonlinearity by means of its nonlinear mapping ability. The distributed collaborative response surface method was applied to handle multi-object multi-discipline analysis, by decomposing one “big” model with hyperparameters and high nonlinearity into a series of “small” sub-models with few parameters and low nonlinearity. Based on the developed method, the blade-tip clearance reliability analysis of an aeroengine high-pressure turbine was performed subject to the creep behaviors of structural materials, by considering the randomness of influencing parameters such as gas temperature, rotational speed, material parameters, convective heat transfer coefficient, and so forth. It was found that the reliability degree of the clearance is 0.9909 when the allowable value is 2.2 mm, and the creep deformation of the clearance presents a normal distribution with a mean of 1.9829 mm and a standard deviation of 0.07539 mm. Based on a comparison of the methods, it is demonstrated that the proposed method requires a computing time of 1.201 s and has a computational accuracy of 99.929% over 10^4^ simulations, which are improvements of 70.5% and 1.23%, respectively, relative to the distributed collaborative response surface method. Meanwhile, the high efficiency and high precision of the presented approach become more obvious with the increasing simulations. The efforts of this study provide a promising approach to improve the dynamic reliability analysis of complex structures.

## 1. Introduction

Creep is induced by the excessive plastic deformation of high-temperature components in an aeroengine, and seriously influences the performance of blade-tip radial running clearance (BTRRC) and aeroengine. Therefore, effectively designing and controlling the BTRRC is significant in the development of high-performance and high-reliability aeroengines [1]. Under aeroengine operation, the BTRRC is variable, with its work status being subject to numerous loads such as heat load, mechanical load, aerodynamic load, and so forth; therefore, the methods and technology of reasonable BTRRC design were the principal concern of [2,3,4]. Most early works on BTRRC design focused on deterministic analysis by adopting the margin method to ensure the safety of the blade tip [3,4]. Unfortunately, this method ignores randomness and uncertainty of impact factors on the BTRRC, such that it is difficult to reflect the actual change rule of the BTRRC. In fact, most impact parameters, such as geometric sizes, material parameters, physical loads, and so on, inherently hold some randomness and uncertainty during fabrication and operation. With respect to the BTRRC, one alternative approach for deterministic analysis is the probabilistic method, which takes account of the randomness of design parameters. Therefore, it is urgent to conduct reliability analysis of BTRRC with respect to the randomness of parameters from a probabilistic perspective, in order to acquire more practical results catering to engineering.

With the development of probabilistic design in structural reliability [5,6,7,8,9], numerous approaches have appeared, mainly consisting of direct simulation methods combined with the Monte Carlo (MC) method [10,11] and the surrogate model approach (also called response surface method, RSM) [12,13]. Although the MC method has high simulation precision in structural reliability analysis subject to precise finite element (FE) models, practical loads, and boundary conditions, the computational efficiency of the MC method finds it difficult to meet the requirements of probabilistic analysis of complex structures with excessive computational burdens due to their complex structure and conditions, such as variable geometry, high nonlinearity, transients, endured loads, and so forth [9]. To address this issue, the surrogate model method, especially quadratic polynomial-based RSM, was developed to improve the efficiency of the probabilistic analysis of complex structures, finding widespread application in many engineering fields [12,13]. However, the RSM is unsatisfying with respect to computing precision due to the approximation of quadratic polynomials to real models. With the development of reliability theory and computer technology, advanced response surface methods have emerged through the integration of advanced algorithms [14,15]. Zhang et al. [16] developed an extremum response surface method (ERSM) in order to overcome the transient problem in the dynamic reliability analysis of flexible mechanisms and validated the ERSM to be highly computationally efficient with acceptable precision. Lu et al. [17] developed an improved ERSM for the reliability sensitivity analysis of multi-failure compressor blisk, by introducing a Kriging model into the ERSM model. The dynamic analysis of BTRRC involves many objects (i.e., disk, blade, casing), multiple disciplines (thermodynamics, aerodynamics, rotodynamics, etc.), transients (time-varying characteristics), hyperparameters (large-scale design variables) and a high degree of nonlinearity. This analysis is a multi-object multi-discipline (MOMD) transient nonlinear analysis with hyperparameters, whereby the computational burden is greatly increased. Therefore, it is difficult to directly adopt the above RSMs for the BTRRC reliability analysis. To address this issue, Bai and Fei [18,19] proposed a distributed collaborative response surface method (DCRSM) to carry out the reliability analysis of an aeroengine high-pressure BTRRC, and illustrated the validity of DCRSM in efficiency and accuracy. Later, to further improve the DCRSM in BTRRC reliability design and turbine blisk reliability evaluation, Fei et al. [20] developed a distributed collaborative extremum response surface method (DCERSM) by incorporating the ERSM concept to handle the transient analytical problem. The investigation in [18,19,20] demonstrated that the DCRSM has potential in MOMD probabilistic analysis with high modeling precision and simulation efficiency, providing useful insights for the purpose of improving the current BTRRC reliability analysis in this paper.

With the development of artificial intelligence, the presence of generalized regression neural network (GRNN) [21] opens the possibility for further enhancing the DCRSM with respect to computing precision and efficiency, because the GRNN has strong nonlinear mapping ability and robustness and small samples, and thus can address highly nonlinear problems, reduce the extraction of samples, and improve the modeling speed. Adel et al. [22] established the GRNN model to predict the wastage of condensers and verified the accuracy of the developed GRNN model. Zhao et al. [23] built the widely used GRNN model to predict the quantity of shipments based on small samples. Machado et al. [24] adopted GRNN to optimize the wavelet transform of voltage signals in electrical power system. Li et al. [25] combined the fruit fly optimization algorithm and GRNN to structure the prediction model of power load. Zhang et al. [26] discussed generalized regression extremum neural network (GRENN) model based on GRNN and ERSM in order to improve the analytical precision of an aeroengine turbine blisk fatigue life reliability analysis. Sun et al. [27] revealed that GRNN had a short training time, good stability and high fitting precision by comparing it to a back propagation neural network for the prediction of air quality. All of the above works show the merits of GRNN with respect to computational accuracy and efficiency. 

To improve the computational efficiency and precision of BTRRC dynamic reliability analysis with many objects, multiple disciplines, high nonlinearity, transients, and hyperparameters, this paper proposes distributed collaborative generalized regression extreme neural network (DCGRENN) method, by incorporating both the highly nonlinear mapping ability of ERSM and the MOMD coordination potential of DCRSM. In the DCGRENN, the GRENN is used to handle the transient problem by simplifying the response process as one extremum value and address the nonlinearity by the nonlinear mapping capacity. The thought of DCRSM is applied to handle MOMD analysis with hyperparameters and high nonlinearity by decomposing the “big” model into a series of “small” sub-models with few parameters and low nonlinearity. The DCGRENN is employed in order to carry out the reliability analysis of turbine BTRRC subject to the creep behaviors of structural materials by considering the randomness of influencing parameters. In addition, the comparison of methods is conducted to validate the DCGRENN. 

The remainder of this paper is organized as follows. The theory and method of DCGRENN are discussed in Section 2, comprising high-temperature creep theory, GRNN, GRENN, DCGRENN and reliability calculation approaches. Section 3 investigates the dynamic reliability analysis of turbine BTRRC with DCGRENN method, including the flowchart of BTRRC reliability analysis with DCGRENN, the finite element (FE) analysis of BTRRC, DCGRENN modeling, BTRRC reliability analysis with DCGRENN and DCGRENN validation. In Section 4, the main conclusions are summarized.

## 2. Theory and Methods

### 2.1. High-Temperature Creep Theory 

**Figure 1:**
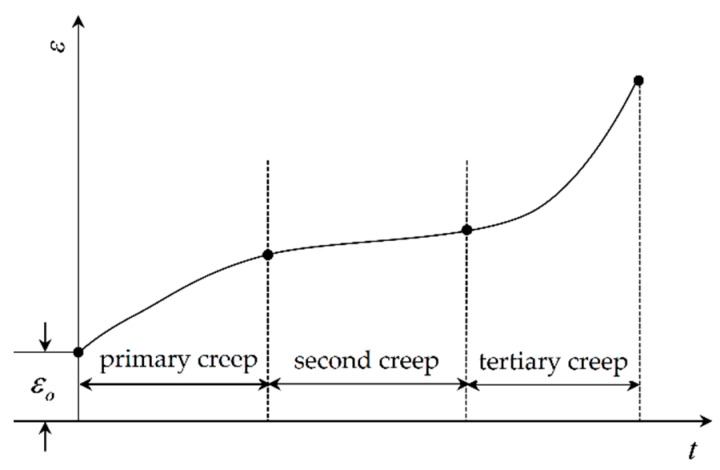
Creep curve of a structure.

In mechanical strength, the creep of a structure is the permanent deformation of aeroengine turbine structures induced by high temperature load when the stress is less than yield strength [28]. Obviously, creep curve describes the variation of the strain *ε* of structural materials with time *t*, as explained in Figure 1.

In engineering, the Norton implicit model is usually adopted to express the second constitutive relation of structural materials [29], i.e.,

(1) Δεcreep=C1σC2exp−C3T

in which ${\Delta \varepsilon }_{creep}$ is creep strain; *C_i_* (*i* = 1, 2, 3) is the parameter of material creep; *T* is test temperature.

Based on the creep tensile experiment of the material GH4133B (Ni-Cr-based precipitation hardening-type deformation high-temperature alloy) under cyclic loading, the creep data are obtained. Based on the least squares method [30], the creep parameters of GH4133B were obtained and are presented in Table 1.

With respect to the obtained creep parameters (*C*_1_, *C*_2_ and *C*_3_), the creep model of GH4133Bis built as presented in Equation (1), and this will be used in the calculation of blade-tip clearance in the investigation below, regarding GH4133B as the material of the BTRRC. 

### 2.2. Generalized Regression Neural Network

**Figure 2:**
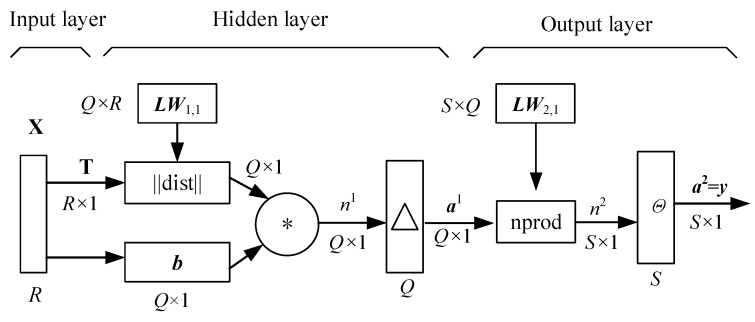
The schematic diagram of the generalized regression neural network.

The generalized regression neural network (GRNN) is a nonlinear regression-based feedforward neural network model, including an input layer, a hidden layer, and an output layer [26]. The GRNN has good ability for nonlinear mapping and achieves satisfactory precision between input parameters and output response when carrying out nonlinear modeling. The network structure of GRNN is illustrated in Figure 2.

In Figure 2, ***X*** and ***T*** are the matrices of input samples and output samples, respectively; *Q × R* indicates the dimensions of matrix ***LW***_1,1_, which is the weighted matrix in the hidden layer, where *Q* and *R* are the numbers of training samples and input parameters, respectively; ||dist|| denotes the Euclidean distance function; ***b*** is the threshold of *Q* neural cells in the hidden layer; *n*^1^ expresses the network vector of the hidden layer; △ indicates the transfer (Gauss) function; ***a***^1^ is the output of the neuro cell; *S × Q* indicates the dimensions of the matrix ***LW***_2,1_, which is the connection threshold value between the hidden layer and the output layer, where *S* is the number of output parameters; nprod is the weight function of the output layer; *n*^2^ indicates the network vector of output layer; *Θ* is the purelin transfer function of the output layer; ***y*** = *a*^2^ explains the outputs of the neuro cell in the output layer. The input layer transmits the sample ***X*** into the hidden layer, in which one nerve cell has one sample and there are *Q* nerve cells. In the process of transmission, the transfer function is a Gauss function with the matrix of weights $LW_{1,1}$ and the threshold level vector ***b***. The third layer is the output layer to which the information is transferred from the hidden layer with respect to the matrix of weights ***LW***_2,1_. Herein, the weight function is the normalized dot product function denoted by nprod.

With respect to the line transfer function $y^{j}=purelin\left(n^{j}\right)$ of the *j*th output *n^j^* of the GRNN model, the mathematical model of GRNN for the response of the *j*th group of training samples is expressed by

(2) $$y^{j}=purelin\left(n^{j}\right)=\frac{LW_{2,1}{\left[a^{j}\right]}^{T}}{\sum_{i=1}^{Q}a_{i}^{j}}$$

where *exp* is the natural exponential function; $a^{j}=\left[a_{1}^{j},a_{2}^{j},\cdots ,a_{i}^{j},\cdots a_{Q}^{j}\right]$ indicates the output vector of *Q* nerve cells corresponding to the *j*th group of input samples, in which $a_{i}^{j}$ (*i* = 1, 2, …, *Q*) is the *i*th element in $a^{j}$.

### 2.3. Generalized Regression Extremum Neural Network Method

**Figure 3:**
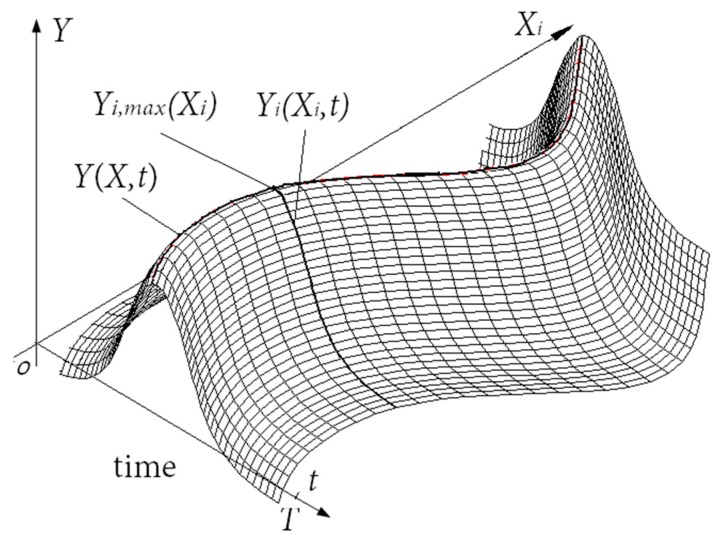
The basic concept of the generalized regression extremum neural network method.

Since dynamic probabilistic analysis is a random process, it is difficult to resolve the probabilistic analysis of complex structures by directly adopting the GRNN method. In this case, the traditional approaches involve fitting a large number of response surface models in the time domain, and then artificially selecting the response at one time point as the probabilistic computing point [20]. However, it is hard for this method to guarantee the precision of the selected computing point in engineering applications. To overcome this issue, a generalized regression extremum neural network (GRENN) method is developed by introducing the thought of extremum response into the GRNN model. The GRENN method simplifies the dynamic output response of numerous response models based on the GRNN method as one extremum value of the response process. In this paper, the maximum output responses of input samples in time domain [0, T] are focused on dynamic probabilistic analysis. The basic concept behind the GRENN method is explained in Figure 3.

In Figure 3, $Y_{i}$($X_{i},t$) is the output response of the *i*th input sample in the time domain [0,T] and $Y_{i,max}\left(X_{i}\right)$ is the maximum of $Y_{i}$($X_{i},t$) in the time domain. When $\left\{Y_{i,max}\left(X_{i}\right)\right\}$ denotes the set comprising the maximum output responses corresponding to all input samples, the fitted extremum response curve Y(X,t) is obtained, as follows:

(3) $$Y\left(X,\;t\right)=f\left(X,\;t\right)=\left\{Y_{i,max}\left(X_{i}\right)\right\},i=1,\;2,\;\ldots ,\;Q$$

where *Q* is the number of samples. 

Based on the mathematical model of GRNN in Equation (2), the mathematical model of GRENN is

(4) $$Y\left(X\right)=Max\left\{Y_{i}\right\}=Max\left\{\frac{LW_{2,1}{\left[a_{i}\right]}^{T}}{\sum_{j=1}^{Q}a_{ij}}\right\},\left(i=1,2,\cdots ,Q\right)$$

in which *i* indicates the *i*th training sample; *j* expresses the *i*th random variable. 

### 2.4. Distributed Collaborative Generalized Regression Extremum Neural Network 

Although the GRENN method holds promise with respect to solving the transient problem in structural dynamic probabilistic analysis, it still poses one challenge in handling the dynamic probabilistic analysis of structural systems involving multiple objects, multiple disciplines, and hyperparameters. If the GRENN is directly applied to the probabilistic analysis of MOMD structure, it is unworkable for effectively dealing with the analysis problem of many objects and multiple disciplines simultaneously. To address this issue, the DCGRENN method is developed by integrating the thoughts of DCRSM [18] and GRENN [26]. Distributed coordinative technique in DCRSM is applied to handle the MOMD analysis by dividing the “big” model with high-nonlinearity and hyperparameters into a series of simple “small” models with low nonlinearity and few parameters. GRENN is used to address the transient problem in dynamic probabilistic analysis by reducing the response process as one extreme value, and to deal with the problem of nonlinearity by through its ability in highly nonlinear mapping. In other words, in MOMD dynamic probabilistic analysis using the DCGRENN method, the whole (“big”) model of the MOMD structure is divided into many single-object single-discipline (SOSD) sub-models (“small” models), so that the probabilistic analysis of the “big” MOMD model is decomposed into a large number of “small” SOSD models. In the process of SOSD probabilistic analysis, the GRENN models of SOSD models are built as distributed GRENNs (DGRENNs). We consider the responses of DGRENN models as the inputs for the collaborative generalized regression extreme neural network (CGRENN) method in order to perform a collaborative dynamic probabilistic analysis of the structure. The collaborative relationship among the extremum outputs of sub-models is addressed for MOMD dynamic probabilistic analysis. Therefore, the distributed collaborative strategy with GRENN is likely to overcome the problem of MOMD analysis, transient response and hyperparameters in the dynamic probabilistic analysis of complex structures, and then to improve the analytical precision and efficiency. 

When one mechanical structure comprises *m* (*m*∈*Z*) objects (substructures) and the analysis of each object involves *n* (*n*∈*Z*) disciplines, with respect to the DC strategy, the complicated MOMD analysis is transferred into a series of simple SOSD analyses. When ***X***^(*pq*)^ is the input sample vector of *q*th discipline in *p*th object, the corresponding output response, *Y*^(*pq*)^ is expressed as

(5) $$Y^{\left(pq\right)}=f\left(X^{\left(pq\right)}\right)\;(p=1,\;2,\;\ldots m;\;q=1,\;2,\;\ldots n)$$

subject to

(6) $$X^{pq}={\left[x_{1}^{pq}\;x_{2}^{pq}\;\ldots \;x_{k}^{pq}\right]}^{T}$$

where *k* is the number of random variables in single discipline.

According to Equation (4), the DGRENN model is defined as

(7) $$Y^{\left(pq\right)}=f\left(X^{\left(pq\right)}\right)=Max\left\{\frac{LW_{2,1}^{pq}{\left[a_{i}^{pq}\right]}^{T}}{\sum_{j=1}^{Q}a_{ij}^{pq}}\right\}$$

in which $LW_{2,1}^{pq},\;LW_{1,1}^{pq}$ and $a_{i}^{pq}=\left[a_{i1}^{pq},a_{i2}^{pq},\ldots ,a_{iQ}^{pq}\right]$ are the weight matrix in the hidden layer, weight matrix in the output layer and the output vector of the output layer in the DGRENN model of disciplines.

Regarding the output responses ${\left\{Y^{\left(pq\right)}\right\}}_{pq=1}^{m,n}$ of the *q*th discipline in the *p*th object as the random input variables $X^{\left(p\right)}$ of the *p*th object model analysis, i.e.,

(8) $$X^{\left(P\right)}={\left\{Y^{\left(pq\right)}\right\}}_{pq=1}^{m,n}$$

When $Y^{\left(p\right)}$ is the response of the *p*th object, the response surface function (DGRENN) of objects is

(9) $$Y^{\left(p\right)}=f\left(X^{\left(p\right)}\right)=\;Max\left\{\frac{LW_{2,1}^{p}{\left[a_{i}^{p}\right]}^{T}}{\sum_{j=1}^{Q}a_{ij}^{p}}\right\}$$

Here, $LW_{2,1}^{p}$, $LW_{1,1}^{p}$ and $a_{i}^{p}=[a_{i1}^{p},\;a_{i2}^{p},\ldots ,a_{iQ}^{p}]$ are the weight matrix in the hidden layer, weight matrix in the output layer and the output vector of the output layer in the DGRENN model of objects.

Similarly, regarding the outputs ${\left\{Y^{\left(p\right)}\right\}}_{p=1}^{m}$ of all of the object analyses as the random input variables $\tilde{X}$ of the whole coordinative model (CGRENN), i.e.,

(10) $$\tilde{X}={\left\{Y^{\left(p\right)}\right\}}_{p=1}^{m}$$

the output response $\tilde{Y}$ of the MOMD overall CGRENN model is

(11) $$\tilde{Y}=\;f\left(\tilde{X}\right)=\;Max\left\{\frac{\tilde{LW_{2,1}}\tilde{{\left[a_{i}\right]}^{T}}}{\sum_{j=1}^{Q}\tilde{a_{ij}}}\right\}$$

where $\tilde{LW_{2,1}}$, $\tilde{LW_{1,1}}$ and $\tilde{a_{i}}=\left[\tilde{a_{i1}},\tilde{a_{i2}},\ldots ,\;\tilde{a_{ij}}\right]$ are the weight matrix in the hidden layer, weight matrix in the output layer and the output vector of the output layer in the CGRENN of the whole MOMD model. 

From the above analysis, the whole GRENN model in Equation (4) is divided into many small GRENN models, such as the DGRENN models in Equations (7) and (9) and the CGRENN model in Equation (11), to address the dynamic probabilistic analysis of complicated MOMD structure. This method is called the DCGRENN method in this paper. 

### 2.5. Reliability Calculation Approaches 

The BTRRC of aeroengine turbines is determined by the radial deformations of the turbine disk, blade and casing and pre-clearance [1,2]. When *Y*_d_(*t*), *Y*_b_(*t*) and *Y*_c_(*t*) are the radial deformations of turbine disk, blade and casings at time *t*, respectively, the deformation of BTRRC *τ*(*t*) [3,4] is

(12) $$\tau \left(t\right)=\;Y_{d}\left(t\right)+Y_{b}\left(t\right)-Y_{c}\left(t\right)$$

When *δ* is the allowable value of steady blade-tip clearance, the BTRRC at time *t* is defined as

(13) $$Y\left(t\right)=\delta -\tau \left(t\right)=\delta -Y_{d}\left(t\right)-Y_{b}\left(t\right)+Y_{c}\left(t\right)$$

When $t=t_{0}$, the BTRRC reaches its minimum. The limit state function of BTRRC (Equation (13)) can be rewritten as

(14) $$Y=\delta -Y_{d}-Y_{b}+Y_{c}$$

in which $Y_{d},\;Y_{b}\;\mathrm{and}\;Y_{c}$ are the radial deformations of the turbine disk, blade and casings at *t*_0_. 

As shown in Equation (14), *Y* > 0 indicates that the BTRRC is smaller than the allowable value, which explains the safety of the assembly or the BTRRC; otherwise, the BTRRC is in failure status. Assuming that the random variables in Equation (14) are mutually independent with the means $\mu =\left[\mu_{d}\;\mu_{b}\;\mu_{c}\right]$ and the standard deviations $\sigma =\left[\sigma_{d}\;\sigma_{b}\;\sigma_{c}\right]$, the expectation and variance of the response *Y* in Equation (14) are

(15) $$\{\begin{array}{c} E=\mu_{Y}\left(\mu_{d},\mu_{b},\mu_{c},\sigma_{d},\;\sigma_{b},\sigma_{c}\right)\\ D=D_{Y}\left(\mu_{d},\mu_{b},\mu_{c},\sigma_{d},\;\sigma_{b},\sigma_{c}\right) \end{array}$$

in which *E* indicates the mean value of blade-tip clearance; *D* denotes the variance of blade-tip clearance; and $\mu_{Y}\left(\cdot \right)$ and $D_{Y}\left(\cdot \right)$ are the mean and variance functions of blade-tip clearance, respectively, which is defined by the means and variances of disk, blade and casing deformations.

The reliability degree of BTRRC (response) can be expressed as

(16) $$R=\Phi \left(\frac{\mu_{Y}}{\sqrt{D_{Y}}}\right)$$

where Φ(·) is the function of accumulative normal distribution. 

## 3. Reliability Analysis of Blade-Tip Radial Running Clearance

**Figure 4:**
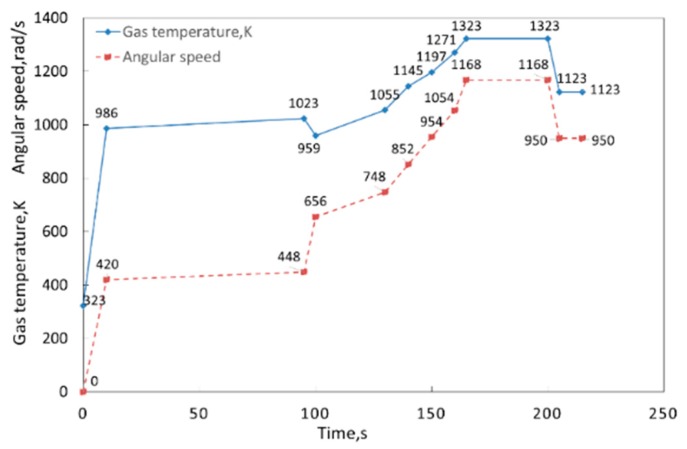
Flight profile of aeroengine.

This paper selects the BTRRC of the first stage of the high-pressure turbine of an aeroengine as the object of study. In line with engineering experience, the flight profile of an aeroengine from start to cruise is considered as the analytical range in the time domain [0, 215 s] [9], in which 12 time points are selected as computing points. The flight profile is displayed in Figure 4. 

**Figure 5:**
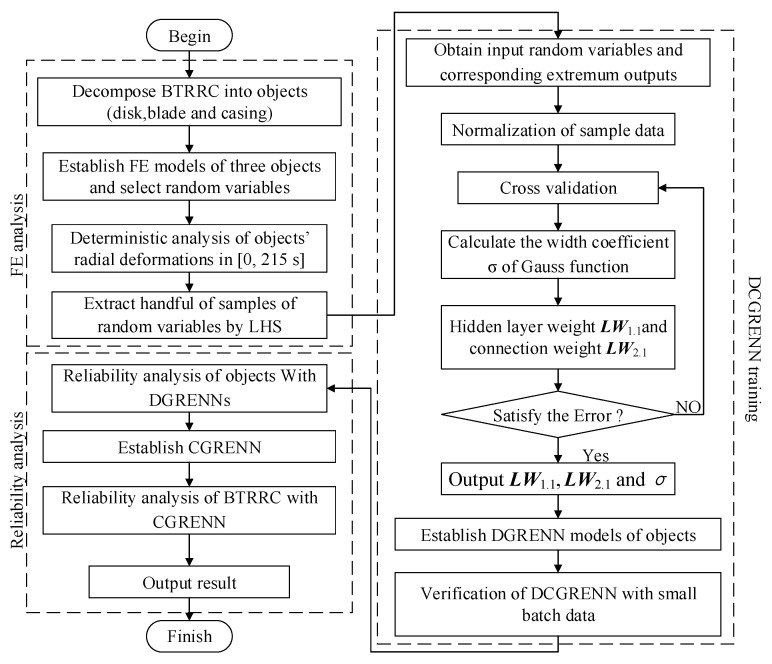
The flowchart of BTRRC reliability analysis with DCGRENN method.

In line with the proposed DCGRENN method, the flowchart of the reliability analysis of BTRRC is structured in Figure 5 and is also summarized as follows:

***Step 1***: Structure the FE models of the turbine disk, blades and casings through the decomposition of the BTRRC analysis problem into the radial creep problems of the three objects (disk, blade and casing);

***Step 2***: Implement the thermal-structural coupling deterministic analysis of the three objects in the time domain [0, 215 s] to acquire the change rule of BTRRC and determine the computing point with the minimum BTRRC by considering the input random variables (i.e., gas temperature, rotor speed, material parameters and convective heat transfer coefficient), the related boundary conditions, and multiple disciplines (i.e., heat load and centrifugal load);

***Step 3***: Extract the handful of samples for the random input variables by Latin hypercube sampling (LHS) technology [17], to perform FE analyses and obtain the creep deformations of the three objects (turbine disk, blade and casings) with respect to the samples, and regard the samples involving input parameters and the maximum values of the output responses as training samples;

***Step 4***: Normalize the training samples to obtain the optimal smooth factors, the weighted values in the hidden layer and the connection weights between the hidden layer and the output layer using the cross-validation method [31], and then structure the distributed generalized regression extremum neural network (DGRENN) models of three objects’ radial creep deformations;

***Step 5***: Check the precision of each object model. Return to ***Step 3*** if the computational accuracy does not satisfy engineering requirements, or continue to ***Step 6***;

***Step 6***: Conduct reliability analyses of the three objects’ radial creep deformations with the DGRENN models to obtain the distribution features of the maximum output responses;

***Step 7***: Establish the collaborative generalized regression extremum response surface (CGRENN) model (i.e., Equation (14)) of BTRRC by adopting coordinative method to coordinate the three DGRENN models;

***Step 8***: Implement the reliability analysis of BTRRC with the required CGRENN model by performing enough simulations and considering the creep deformations of the turbine disk, blades and casings as random input variables.

### 3.1. Finite Element Analysis

#### 3.1.1. Finite Element Modeling

**Figure 6:**
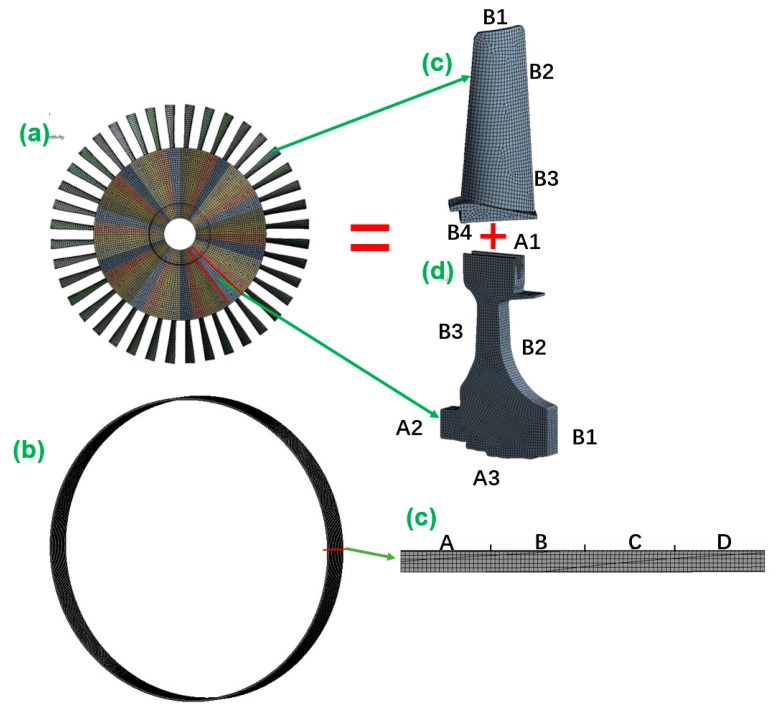
Finite element models.

As shown in Figure 6, we decomposed the BTRRC structure into the structures of the turbine blade (Figure 6c), the disk (Figure 6d) and the casings (Figure 6b,e) [14], with respect to the DCGRENN method. Accordingly, the analysis of BTRRC analysis can be transformed into analyses of three objects’ deformations. Just like in Figure 6, the FE models of the three objects were built and meshed with hexahedral elements to generate 66,399 nodes and 16,413 cells for the disk, 26,266 nodes and 7333 cells for the blade and 66,183 nodes and 9180 cells for the casing. The turbine disk contains cooling holes and is cooled in operation. The symbols A1, A2, A3, B1, B2, and B3 indicate the different positions on the disk. The disk model was a simplified mortise structure and underwent symmetrical loads and boundary conditions. The mortise and cooling holes of the blade structure were ignored. B1, B2 and B3 denote the different areas of the blade. Due to the sensitivity of the casing bush ring on the deformation of casings and BTRRC, the bush ring was considered as the analyzed object to investigate casing creep deformation, in which the case model was divided into four segments: A, B, C and D.

#### 3.1.2. Selection of Random Variables 

In this paper, many parameters, such as rotational speech *ω*, gas temperature *T*, heat conductivity coefficient *λ*, elasticity modulus *E*, convective heat transfer coefficient *α*, and material density *ρ*, are considered to be random input variables. These are assumed to be mutually independent and to follow normal distributions. The distribution characteristics of the variables are listed in Table 2. The temperatures and convective heat transfer coefficients on different positions on disk, blade and casing are computed according to the heat exchange between structures and gas temperature [14,18]. In the variables of the disk, the subscripts a1, a2, a3, b1 and b2 of *T* stand for the temperatures on the areas of A1, A2, A3, B1 and B2, respectively, and the subscripts d1, d2 and d3 of *α* indicate the convective heat transfer coefficients on B1, B2 and B3 on the disk model, respectively. For the variables of the blade, the subscripts b1, b2, b3 and b4 are the locations B1, B2, B3 and B4 on the blade model, respectively. In the variables of the casing, *T*_i_ and *T*_o_ are the temperatures inside and outside the casing rings respectively, and the subscripts c1, c2, c3, c4 and o of *α* indicate the convective heat transfer coefficients on segments A, B, C and D and the outside of casing ring.

#### 3.1.3. Deterministic Analyses of Three Objects

**Figure 7:**
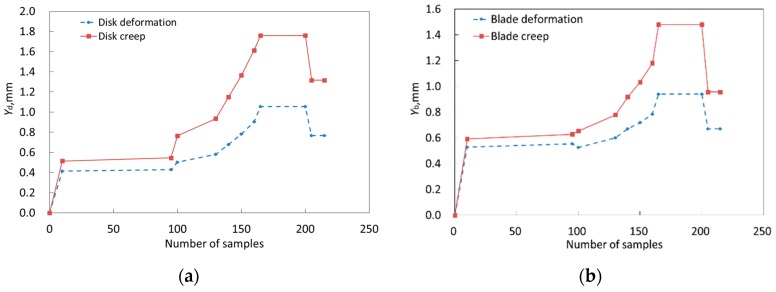

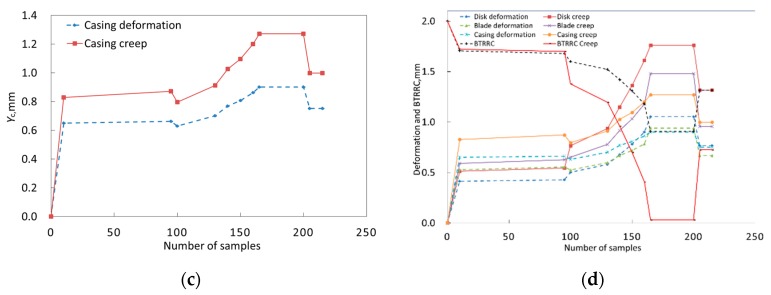
The change of objects’ deformations and BTRRC: (**a**) disk, (**b**) blade, (**c**) casing, (**d**) BTRRC.

In the deterministic analysis of aeroengine BTRRC, the aerodynamic load was ignored because it is not significant in terms of its contribution to the stress for the turbine disk and casing, in comparison to the temperature load and the centrifugal load [32]. With reference to the flight profile of the aeroengine, the loads of heat and centrifugation and the nonlinearity of thermal expansivity, the means of variables in Table 2 were imported into the FE models in order to perform the thermo-structural coupling analysis of three objects under normal status, and to carry out the thermo-structural coupling analysis under creep. For the two analyses, the change curves of the disk, blade and casing deformations *Y*_d_(*t*), *Y*_b_(*t*) and *Y*_c_(*t*) with time are shown in Figure 7a–c. When the static blade-tip clearance *δ* = 2.0 mm, in line with Equations (12) and (13), the variation of BTRRC *Y*(*t*) with time are required in Figure 7d.

**Figure 8:**
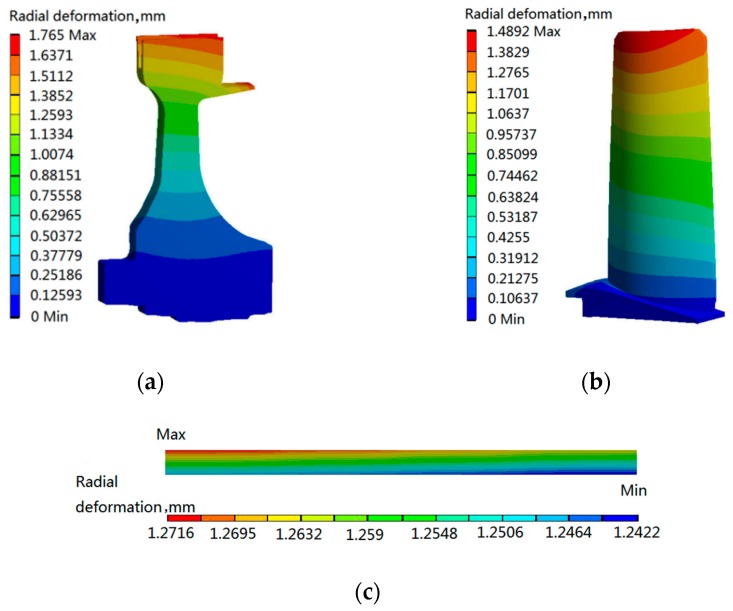
Distributions of creep deformations of disk, blade and casing at *t* = 180 s: (**a**) disk, (**b**) blade, (**c**) casing.

As illustrated in Figure 7, the creep seriously influences the BTRRC of aeroengine, and the BTRRC firstly decreases from start to cruise for the aeroengine. At *t* = 180 s, the BTRRC reaches its minimum and increases a bit when the aeroengine starts to cruise. Obviously, the dangerous point with respect to BTRRC occurs at *t* = 180 s, at which point gas temperature and rotational speech reach their maximum. The creep deformations of three objects at *t* = 180 s are shown in Figure 8.

As revealed in Figure 8, at *t* = 180 s, the maximum creep deformations of the turbine disk, blade and casing were 1.765 mm, 1.4892 mm and 1.2716 mm. At that point in time, the maximum creep deformation of BTRRC is 1.9826 mm. Therefore, *t* = 180 s was selected as the computing point for BTRRC reliability analysis in this paper.

### 3.2. Modeling of the Distributed Collaborative Generalized Regression Extremum Neural Network

With respect to the distribution features of random variables in Table 2 and FE models, 150 samples for the maximum deformations of turbine disk, blade and casing were extracted by LHS technology as the pool of samples. From the pool of samples, 120 samples were selected as the training samples to establish the DGRENN models, and the remainder (30 samples) were the test samples for checking the developed DGRENN models.

**Figure 9:**
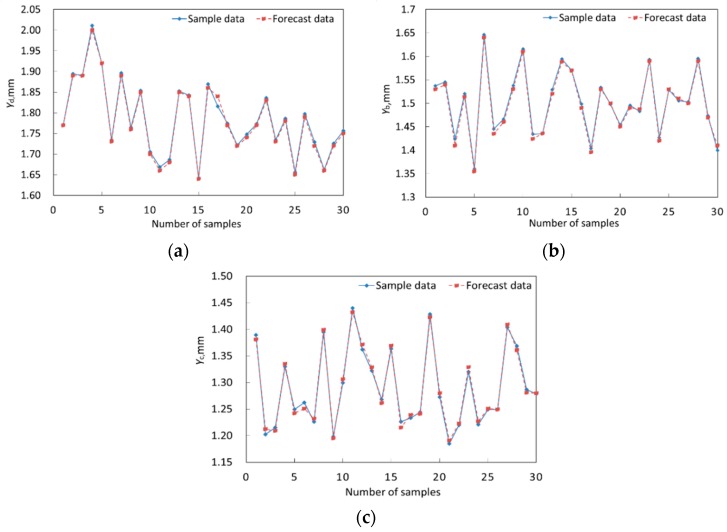
Comparison of prediction results with samples values (original data). (**a**) Disk, (**b**) blade, (**c**) casing.

Along with the theory and concept of the DCGRENN method and BTRRC reliability analysis, in the GRENN model, the Gauss function is selected as the transfer function in the hidden layer, in which the weights matrix ***LW***_1,1_ was computed using the Euclidean distance function [33,34,35]. Then the outputs of the hidden layer were regarded as the connected weights matrix ***LW***_2,1_ of the hidden layer and the output layer. To avoid excessive modeling error, the data of the acquired samples were normalized. The normalized data were applied in order to train the DGRENN model by computing the parameters of DGRENN using the cross-validation method [31]. Finally, smooth factors of $\sigma_{d}$ = 0.87, $\sigma_{b}$ = 0.84 and $\sigma_{c}$ = 0.76 were obtained, the weights matrix ***LW***_1,1_ in the hidden layers of three objects (*Y*_d_, *Y*_b_ and *Y*_c_), the weight matrix ***LW***_2,1_ in the output layer, and the threshold matrix **b**, which are illustrated in Appendix A. By introducing the Appendix A, comprising three equations, into Equation (9), the DGRENN models were structured. The remaining 30 samples were employed to check the established DGRENN model. The prediction results are shown in Figure 9, and their root-mean-square errors (RMSEs) are listed in Table 3. It is revealed in Figure 9 and Table 3 that the predicted results are almost consistent with the original data due to small RMSE, indicating the effectiveness and accuracy of the presented DGRENN models.

**Figure 10:**
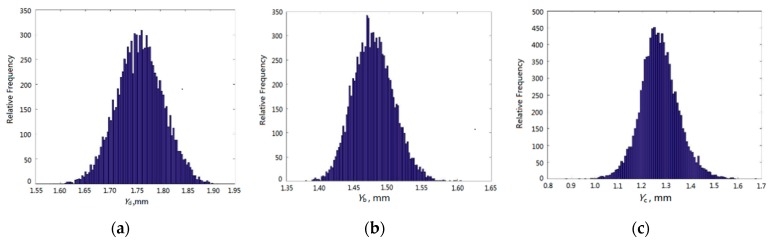
The histograms of radial creep deformations of disk, blade and casing. (**a**) Disk creep deformation; (**b**) blade creep deformation; (**c**) casing creep deformation.

The DGRENN models, instead of the FE models, were simulated 10^4^ times using the MC method in order to complete the dynamic probabilistic analysis of the three objects (disk, blade and casing) and then to acquire the creep deformations of objects. The histograms of creep deformations are shown in Figure 10 and the distribution features of *Y*_d_, *Y*_b_ and *Y*_c_ are displayed in Table 4.

### 3.3. Creep-Based Reliability Analysis

**Figure 11:**
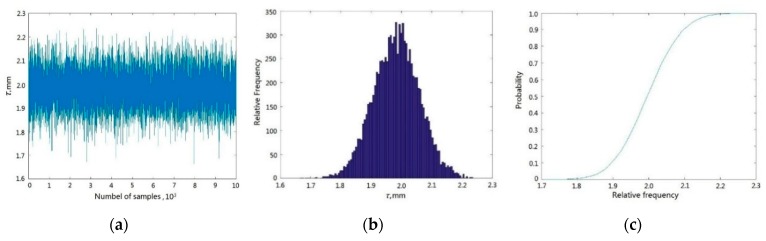
The results of BTRRC reliability analysis using the DCGRENN method. (**a**) History simulation graph of *τ*, (**b**) histogram of *τ*, (**c**) cumulative probability graph of *τ*. Note: *τ*—the deformation of BTRRC.

In line with the DCGRNN method, the creep deformations of three objects were considered as the inputs of BTRRC reliability analysis. Equations (12) and (14) are regarded as the CGRENN models of BTRRC probabilistic analysis with 10^4^ simulations using the MC method. Based on this analysis, the history simulation graph, histogram and probabilistic cumulative graph of the maximum creep deformation of BTTRC at *t* = 180 s are shown in Figure 11.

As shown in Figure 11, the mean and standard deviation of BTRRC are 1.9829 mm and 0.07539 mm, respectively; the reliability degree of BTRRC is determined as 0.990 9 due to 91 failed simulations; and the analysis requires 1.216 s with a static blade-tip clearance *δ* = 2.2 mm. On the basis of engineering experience, these results are basically appropriate for BTRRC design and control.

### 3.4. Validation of Method

To validate the DCGRENN method, the reliability analyses of BTRRC were conducted using the MC method, the distribution collaborative response surface method (DCRSM) [18], and the DCGRENN method, in turn, under the same simulation conditions with random variables presented in Table 2 and using a computer with an Intel^®^ Core™ i7-8400 processor and 16 GB RAM (Random Access Memory). The computing precision and the time required by the three methods were assessed under different simulations (10^2^, 10^3^, 10^4^, 10^5^ and 10^6^) with static blade-tip clearance *δ* = 2.2 mm. The analytical results are listed in Table 5 and Table 6. The precision of BTRRC reliability degree was regarded to evaluate the precision of the methods, with the MC method acting as a reference in BTRRC reliability analysis. With respect to the 10^4^ simulations, the computing precision $D_{P}$ in Table 6 is defined by

(17) $$D_{P}=1-\frac{\gamma_{a}-\gamma_{m}}{\gamma_{a}}\times 100\%$$

where $\gamma_{a}$ is the reliability degree computed by MC method; $\gamma_{m}$ is the reliability degree calculated by DCRSM or DCGRENN.

As seen in Table 5: (1) the computing time of the three method increases with the increase in the number of simulations, owing to the increase in the computing loads, wherein the increase for the DCGRENN method is slower than that of the other two methods. For instance, from 10^2^ simulations to 10^6^ simulations, the DCGRNN method only increased about two-fold, while the DCRSM increased more than 100-fold. Additionally, it is amazing that the MC method increased more than 100-fold from 10^2^ simulations to 10^4^ simulations; (2) DCRSM and DCGRENN required far less time that the MC method to carry out the same number of simulations, because surrogate model-based simulation is easier and faster than FE-based simulation. This is why the surrogate model was used to perform the reliability analysis of BTRRC in this study; (3) the MC method cannot conduct the probabilistic analysis of BTRRC when the number of simulations is larger than 10^4^, due to the unaffordable computational burden. In this case, the results of 10^4^ FE simulations were used as a reference for assessing the computational time and efficiency of the other methods in this study, because the credibility of reliability analysis is positively correlated the number of FE simulations in engineering reliability design; (4) the developed DCGRENN method showed greater computational efficiency than DCRSM, and the strength of the DCGRENN method was more obvious with the increase in the number of simulations, since the speed of GRNN in simulation is a result of its advantage of a small number of samples relative to quadratic polynomials.

As revealed in Table 6, the computing precision of DCGRENN method is higher than DCRSM, and improved by 1.23% over 10^4^ simulations. It should be noted that the DCGRNN method is better able to ensure computing precision for small simulations. For example, the DCGRENN method is improved by 2.017% at 100 simulations. The reason for this is that the GRENN has good properties with respect to the handling of highly nonlinear and transient problems in structural dynamic probabilistic analysis, and the DC strategy is able to process MOMD analysis problems for complicated structures by reducing the nonlinearity.

In conclusion, the proposed DCGRENN method is able to improve the precision and efficiency of the reliability analysis of complex structures, in addition to the BTRRC, in aeroengines.

## 4. Conclusions

The aim of this paper was to develop a distributed collaborative generalized regression extremum neural network (DCGRENN) method by integrating the generalized regression extremum neural network with nonlinear fitting ability and a small numbers of samples with the distributed collaborative response surface method with multi-object multi-discipline coordinative capability. The developed approach is applied in order to improve the efficiency and precision of the simulations for the purpose of high-pressure blade-tip radial running clearance reliability analysis subject to creep behavior. The main conclusions are summarized as follows:

From the deterministic analysis of blade-tip clearance with creep, it is illustrated that the maximum creep deformations of the turbine disk, blade and casing were 1.765 mm, 1.4892 mm and 1.2716 mm at *t* = 180 s. At this moment, the maximum creep deformation of the blade-tip clearance was 1.9826 mm. This study selected *t* = 180 s as the computing point of the blade-tip clearance reliability analysis. The creep-based dynamic reliability analysis of the blade-tip clearance reveals that the creep deformation of blade-tip clearance obeys the normal distribution with the mean of 1.9829 mm and the standard deviation of 0.07539 mm, and the reliability degree of the blade-tip clearance was 0.9909 when the steady blade-tip clearance *δ* was 2.2 mm, which provides a significant reference for blade-tip clearance design in engineering.

With respect to computational efficiency, the method developed in this study was validated to have high computational efficiency in the dynamic probabilistic analysis of complex structures, thanks to the strengths of the generalized regression extremum neural network and the distributed collaborative strategy. With respect to computing precision, the proposed method was higher by 1.23% than the distributed collaborative response surface method under 10^4^ simulations in blade-tip clearance reliability analysis, owing to the capabilities of the generalized regression extremum neural network in terms of addressing highly nonlinear analysis problems.

## Figures and Tables

**Table 1:** The creep parameters of GH4133B material.

Coefficient	*C*_1_	*C*_2_	*C*_3_
Creep value	8.892 × 10^−13^	7.436	1.267

**Table 2:** The distributions of the random input variables.

Disk			Blade			Casing		
Random Variable	Mean *μ*	Standard Deviation	Random Variable	Mean *μ*	Standard Deviation	Random Variable	Mean *μ*	Standard Deviation
*T*_a1_, K	813	24.39	*T*_b1_, K	1303	39.09	*T*_i_, K	1323	39.69
*T*_a2_, K	483	14.49	*T*_b2_, K	1253	37.59	*T*_o_, K	593	17.79
*T*_a3_, K	473	14.19	*T*_b3_, K	1093	32.79	*α*_c1_, W·m^−2^·K^−1^	6000	180.00
*T*_b1_, K	518	15.54	*T*_b4_, K	813	24.39	*α*_c2_, W·m^−2^·K^−1^	5400	162.00
*T*_b2_, K	593	17.79	*α*_b1_, W·m^−2^·K^−1^	11,756	352.68	*α*_c3_, W·m^−2^·K^−1^	4800	144.00
*α*_d1_, W·m^−2^·K^−1^	1527	45.81	*α*_b2_, W·m^−2^·K^−1^	8253	247.59	*α*_c4_, W·m^−2^·K^−1^	4200	126.00
*α*_d2_, W·m^−2^·K^−1^	1082	32.46	*α*_d3_, W·m^−2^·K^−1^	6547	196.41	*α*_o_, W·m^−2^·K^−1^	2600	78.00
*α*_d3_, W·m^−2^·K^−1^	864	25.92	*α*_d4_, W·m^−2^·K^−1^	3130	93.90	*ρ*, kg·m^−3^	8210	246.3
*ρ*, kg·m^−3^	8210	246.3	*ρ*, kg·m^−3^	8210	246.3	*E*, MPa	163,000	4890
*E*, MPa	163,000	4890	*E*, MPa	163,000	4890	*λ*, W·m^−1^·C^−1^	23.7	0.711
*λ*, W·m^−1^·C^−1^	23.7	0.711	*λ*, W·m^−1^·C^−1^	23.7	0.711			
*ω*, rad·s^−1^	1168	35.04	*ω*, rad·s^−1^	1168	35.04			

**Table 3:** DGRENN prediction evaluation with 30 testing samples.

Object	Number of Test Samples	RMSE, ×10^−4^
Disk	30	6.32
Blade	30	3.61
Casing	30	4.73

**Table 4:** The distribution of the maximum creep deformations of disk, blade and casing.

Distribution Feature	*Y*_d_	*Y*_b_	*Y*_c_
Mean, ×10^−3^ m	1.7591	1.4774	1.2701
Stand deviation, ×10^−5^ m	4.6693	2.9457	8.0059
Distribution	Normal	Normal	Normal

**Table 5:** Computing time of different methods under different numbers of simulations.

Method	Number of Simulations				
	10^2^	10^3^	10^4^	10^5^	10^6^
MC method	10080 s	111600 s	1330560 s	—	—
DCRSM	1.185 s	1.264 s	4.071 s	16.74 s	141.34 s
DCGRENN	1.176 s	1.186 s	1.201 s	1.451 s	2.449 s

**Table 6:** Precision of different reliability methods under different numbers of simulations.

Number of Simulations	Reliability Degree			$\mathrm{Precision}\;D_{P},\;\%$		Improved
	MC Method	DCRSM	DCGRENN	DCRSM	DCGRENN	Precision, %
10^2^	0.99	0.97	0.99	97.822	99.839	2.017
10^3^	0.992	0.978	0.994	98.628	99.758	1.130
10^4^	0.9916	0.9787	0.9909	98.699	99.929	1.230
10^5^	—	0.9793	0.9898	98.759	99.818	1.059
10^6^	—	0.9779	0.9932	98.618	99.839	1.221

## Acronyms

Acronyms	Explanations
BTRRC	Blade-tip radial running clearance
MC	Monte Carlo
RSM	Response surface method
ERSM	Extremum response surface method
DC	Distributed collaborative
DCRSM	Distributed collaborative response surface method
DCERSM	Distributed collaborative extremum response surface method
GRNN	Generalized regression neural network
GRENN	Generalized regression extremum neural network
CGRENN	Collaborative generalized regression extreme neural network
DGRENN	Distributed generalized regression extreme neural network
DCGRENN	Distributed collaborative generalized regression extreme neural network
FE	Finite element
LHS	Latin hypercube sampling
RMSE	Root-mean-square error
SOSD	Single-object single-discipline
MOMD	Multi-object multi-discipline
RAM	Random access memory

## Nomenclature

△ε_creep_	Creep strain
*ε*	Strain
*t*	Time
*T*	Test temperature
*C*_1_, *C*_2_, *C*_3_	Coefficients of material creep
*exp*	Nature exponential function
***X***	Matrix of input samples
*Q*	Number of training samples
*R*	Dimension number of input parameters
*S*	Dimension number of output parameters
***LW***_1.1_	Weighted matrix in hide layer
*Q* × *R*	Dimensions of matrix ***LW***_1.1_
||dist||	Weight (Euclidean distance) function in hide layer
△	Transfer (Gauss) function
*n* ^1^	Network vector in hide layer
***a*** ^1^	Output of neuro cell in hide layer
***LW***_2.1_	Connection threshold value between hide layer and output layer
*S* × *Q*	Stands for the dimensions of matrix ***LW***_2.1_
nprod	Weight function of output layer
	Purelin transfer function of output layer
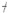
***b***	Threshold level vector
*n* ^2^	Network vector of output layer
*a* ^2^	Output of neuro cell in output layer
*y*	Output of neuro network
***a*** *^j^*	Output vectorof *Q* nerve cells for *j*th group of input samples
$a_{i}^{j}$	The *i*th element in $a^{j}$
*E*	Mean value of blade-tip clearance
*D*	Variance of blade-tip clearance
*μ_Y_*(⋅)	Mean function
$\mu_{d}$	Disk mean value
$\mu_{b}$	Blade mean value
$\mu_{c}$	Casing mean value
$\sigma_{d}$	Disk variance value
$\sigma_{b}$	Blade variance value
$\sigma_{c}$	Casing variance value
*D_Y_*(⋅)	Variance function
*R*	Reliability
Φ(⋅)	Accumulative function
*ω*	Rotational speech
*λ*	Heat conductivity coefficient
*E*	Elasticity modulus
*α*	Convective heat transfer coefficient
*ρ*	Material density
*σ*	Smooth factors
*δ*	Allowable value of steady blade-tip clearance
*γ_a_*	Reliability degree computed by MC method
*γ_m_*	Reliability degree calculated by DCRSM or DCGRENN
$D_{P}$	Computing precision
*Y_i_*	Output response of *i*th input sample in time domain [0, *T*]
*Y_i_,_max_*	The maximum of *Y_i_*(*X_i_*,*t*) in time domain
*i*	Number of training samples
*j*	Number of random variables
*X^(pq)^*	Input sample vector of *q*th discipline in *p*th object
*Y^(pq)^*	Output sample vector of *q*th discipline in *p*th object
$LW_{1,1}^{pq}$	Weight matrix in hidden layer of output layer in the DGRENN model
$LW_{2,1}^{pq}$	Weight matrix in output layer of output layer in the DGRENN model
$a_{i}^{pq}$	Output vector of output layer in the DGRENN model
*X^(p)^*	Input random variables of *p*th object model
*Y^(q)^*	Response of *p*th object
$LW_{1,1}^{p}$	Weight matrix in hidden layer of output layer in the DGRENN model
$LW_{2,1}^{p}$	Weight matrix in output layer of output layer in the DGRENN model
$a_{i}^{p}$	Output vector of output layer
$\tilde{X}$	Input random variables of the whole coordinative model
$\tilde{Y}$	Output response of MOMD overall CGRENN model
$\tilde{LW_{1,1}}$	Weight matrix in hidden layer of output layer
$\tilde{LW_{2,1}}$	Weight matrix in output layer of output layer
$\tilde{a_{i}}$	Output vector of output layer in the CGRENN
$Y_{d}$	Radial deformations of turbine disk
$Y_{b}$	Radial deformations of turbine blade
$Y_{c}$	Radial deformations of turbine casings
*τ*(*t*)	Deformation of BTRRC

## Appendix A

(A1) $$Y_{d}:\{\begin{array}{cc} LW_{1,1} & ={\left[\begin{array}{cccccc} -0.45583 & -0.99293 & \cdots & -0.35689 & 0.80918 & 0.18021\\ -0.49823 & 0.15901 & \cdots & -0.09540 & -0.31448 & -0.96466\\ -0.83687 & 0.03546 & \cdots & -0.54609 & 0.08510 & 0.65957\\ 1.00000 & -0.16312 & \cdots & -0.36170 & 0.90780 & 0.56737\\ 0.25266 & 0.50177 & \cdots & 0.08896 & 1.00000 & -0.88612\\ 0.87857 & 0.15000 & \cdots & 0.76428 & 0.30714 & 0.64285\\ 0.74647 & -0.04225 & \cdots & -0.01408 & 0.97183 & -0.77464\\ 0.78102 & -0.31386 & \cdots & -0.89051 & 0.86131 & 0.24817\\ 0.45774 & -0.67605 & \cdots & -0.69718 & -0.83802 & -0.52112\\ -0.66911 & 0.94117 & \cdots & -1.00000 & 0.83823 & -0.51470\\ 0.07420 & -0.75971 & \cdots & -0.23674 & -0.01766 & 0.41342\\ -0.05839 & 0.43065 & \cdots & 0.97080 & 0.93430 & -0.19708\\ 0.83098 & 0.14788 & \cdots & -0.81690 & -0.19014 & 0.20422\\ -0.40141 & -0.50000 & \cdots & -0.07042 & -0.35915 & -0.28169 \end{array}\right]}_{14\times 120}^{T}\\ LW_{2,1} & ={\left[\begin{array}{cccccc} -0.17501 & -0.20680 & \cdots & -0.50255 & 0.18506 & -0.65902 \end{array}\right]}_{1\times 120}\\ & b={\left[\begin{array}{cccccc} 0.95701 & 0.95701 & \cdots & 0.95701 & 0.95701 & 0.95701 \end{array}\right]}_{1\times 120}^{T} \end{array}$$

(A2) $$Y_{b}:\{\begin{array}{cc} LW_{1,1} & ={\left[\begin{array}{cccccc} -0.41892 & -0.13513 & \cdots & -0.28378 & 0.95945 & 0.79729\\ -0.87837 & 0.89189 & \cdots & -0.77027 & -0.86486 & -0.09459\\ 0.98657 & 0.59731 & \cdots & 0.03355 & -0.20805 & -0.54362\\ -0.78378 & 0.63513 & \cdots & 0.87837 & 1.00000 & 0.48648\\ 0.20270 & -0.62162 & \cdots & -0.52702 & 0.83783 & -0.40540\\ 0.59459 & 0.64864 & \cdots & 0.44594 & 0.41891 & -0.72972\\ 0.65100 & -0.07382 & \cdots & 0.39597 & 0.19463 & -0.59731\\ -0.67785 & 0.55704 & \cdots & -0.89261 & 0.31543 & -0.47651\\ -0.04761 & 0.64625 & \cdots & -0.22448 & 0.52380 & 0.03401\\ -0.17567 & -0.44594 & \cdots & 0.33783 & -0.32432 & -0.39189\\ -0.80331 & -0.79582 & \cdots & -0.82799 & -0.79229 & -0.84122\\ 0.12925 & 0.03401 & \cdots & -0.89115 & -0.08843 & 0.11564\\ 0.51677 & 0.98657 & \cdots & -0.27516 & 0.61073 & -0.58389\\ -0.98657 & -0.14093 & \cdots & 0.89261 & 0.93288 & 0.78523 \end{array}\right]}_{13\times 120}^{T}\\ LW_{2,1} & ={\left[\begin{array}{cccccc} -0.36799 & 0.70122 & \cdots & -0.10947 & 0.59808 & -0.51289 \end{array}\right]}_{1\times 120}\\ & b={\left[\begin{array}{cccccc} 0.99119 & 0.99119 & \cdots & 0.99119 & 0.99119 & 0.99119 \end{array}\right]}_{1\times 120}^{T} \end{array}$$

(A3) $$Y_{c}:\{\begin{array}{cc} LW_{1,1} & ={\left[\begin{array}{cccccc} -0.98657 & -0.14093 & \cdots & 0.89261 & 0.93288 & 0.78523\\ 0.21621 & 0.89189 & \cdots & 0.74324 & -0.24324 & -0.09459\\ 0.32885 & 0.59731 & \cdots & -0.73154 & 0.78523 & -0.54362\\ -0.38255 & 0.62416 & \cdots & -0.87919 & -0.16778 & 0.47651\\ -0.95973 & -0.61073 & \cdots & -0.28859 & -0.62416 & -0.39597\\ 0.86577 & 0.63758 & \cdots & 0.24832 & 1.00000 & -0.73154\\ -0.57534 & -0.09589 & \cdots & -0.69863 & 0.15068 & -0.63013\\ -0.31081 & 0.56756 & \cdots & 0.63513 & -0.97297 & -0.47297\\ 1.00000 & 0.63758 & \cdots & 0.75838 & 0.61073 & 0.03355\\ -0.02013 & -0.44966 & \cdots & 0.73154 & 0.27516 & -0.39597\\ 0.12162 & 0.39189 & \cdots & 0.85135 & -0.56756 & -1.00000\\ -0.14093 & 0.03355 & \cdots & -0.46308 & -0.93288 & 0.11409\\ 0.55704 & 0.98657 & \cdots & 0.10067 & -0.70469 & -0.58389\\ 0.83892 & 0.86577 & \cdots & -0.95973 & -0.51677 & -0.66442 \end{array}\right]}_{14\times 120}^{T}\\ LW_{2,1} & ={\left[\begin{array}{cccccc} 0.05941 & 0.44877 & \cdots & 0.36002 & -0.37608 & -0.58780 \end{array}\right]}_{1\times 120}\\ & b={\left[\begin{array}{cccccc} 1.28093 & 1.28093 & \cdots & 1.28093 & 1.28093 & 1.28093 \end{array}\right]}_{1\times 120}^{T} \end{array}$$
